# Clinical characteristics of patients presenting with depressive states who are not receiving antidepressants: A multicenter observational study in Japanese psychiatric practice

**DOI:** 10.1002/pcn5.70344

**Published:** 2026-05-22

**Authors:** Hiroyuki Muraoka, Ryota Hashimoto, Kentaro Fukumoto, Fumitoshi Kodaka, Kazutaka Ohi, Ken Inada, Norio Yasui‐Furukori

**Affiliations:** ^1^ Department of Psychiatry Kitasato University School of Medicine Sagamihara Japan; ^2^ Department of Pathology of Mental Diseases National Institute of Mental Health, National Center of Neurology and Psychiatry Kodaira Japan; ^3^ Department of NeuropsychCtry, School of Medicine Iwate Medical University Yahaba Japan; ^4^ Department of Psychiatry The Jikei University School of Medicine Tokyo Japan; ^5^ Department of Psychiatry Gifu University Graduate School of Medicine Gifu Japan; ^6^ Department of Psychiatry Dokkyo Medical University School of Medicine Mibu Japan

**Keywords:** antidepressants, clinical decision‐making, depression, diagnostic certainty, psychiatric practice

## Abstract

**Aim:**

To describe clinical characteristics associated with antidepressant non‐use among patients presenting with depressive states in routine psychiatric practice and to explore factors associated with antidepressant prescription among patients with high diagnostic certainty for major depressive disorder (MDD).

**Methods:**

This multicenter cross‐sectional observational study used retrospectively collected routine clinical data from 35 psychiatric facilities in Japan. Eligible patients were those who attended participating facilities during March 2023 and were considered by their treating psychiatrist to be under clinical management for a depressive presentation or depressive episode at the index clinical assessment. All variables, including antidepressant use, current clinical state, symptom severity, and diagnostic certainty, were assessed at that single time point. At each site, when a patient not receiving antidepressants was identified, the next consecutively seen patient receiving antidepressants was enrolled as a comparator to facilitate contemporaneous between‐group comparison. Physicians completed a standardized case report form including demographics, current clinical state, depressive symptom severity, and diagnostic certainty ratings for MDD and alternative diagnoses. Group comparisons were performed in the full cohort. Analyses restricted to patients with high diagnostic certainty for MDD were prespecified as exploratory, and a multivariable logistic regression model was fitted within this subgroup to examine factors associated with antidepressant prescription.

**Results:**

A total of 1342 patients were enrolled (antidepressant use, *n* = 669; no antidepressant use, *n* = 673). High diagnostic certainty for MDD was less frequent in the no‐antidepressant group than in the antidepressant group (22.1% vs. 38.5%). In the no‐antidepressant group, commonly reported reasons for non‐prescription included lack of diagnostic certainty (74.0%), clinical stability or absence of current symptoms (60.3%), and concerns about adverse effects (17.7%) (multiple responses allowed). In exploratory analyses restricted to patients with high diagnostic certainty for MDD (*n* = 400), remitted status and less severe symptom profiles were more frequent in the no‐antidepressant group. In the multivariable model within this subgroup, severe or very severe symptoms (vs. no symptoms) were associated with higher odds of antidepressant prescription (odds ratio [OR] = 4.317, 95% CI 1.067–17.462, *p* = 4.0 × 10^−2^), whereas age, sex, current state, and developmental disorder certainty were not statistically associated.

**Conclusion:**

In this multicenter observational sample, antidepressant non‐use among patients presenting with depressive states was associated with lower diagnostic certainty for MDD and with lower current symptom burden at the time of observation. Among patients with high diagnostic certainty for MDD, greater symptom severity was associated with antidepressant prescription. These findings may help contextualize conservative treatment decisions under diagnostic uncertainty in routine psychiatric practice.

## INTRODUCTION

Major depressive disorder (MDD) is one of the most common and disabling psychiatric disorders worldwide and is associated with substantial functional impairment and health care burden.^1^, ^2^, ^3^, ^4^, ^5^ Antidepressants remain a core treatment option for moderate to severe depressive episodes, and large meta‐analytic evidence supports their efficacy in acute adult MDD.^1^, ^2^, ^3^, ^4^, ^6^, ^7^ At the same time, real‐world prescribing is heterogeneous and often departs from simplified assumptions derived from guideline‐oriented care pathways.^1^, ^2^, ^3^, ^7^, ^8^, ^9^, ^10^, ^11^, ^12^

Population‐level prescribing studies have shown substantial temporal and geographic variation in antidepressant use, but such data do not by themselves explain why a specific patient presenting with depressive symptoms is or is not prescribed an antidepressant at a given clinical encounter.^8^, ^9^, ^10^, ^11^, ^12^ In everyday psychiatric practice, treatment decisions are made under uncertainty and often depend on symptom severity, illness phase, prior treatment response, adverse‐effect concerns, patient preference, and the availability of non‐pharmacological support.^1^, ^2^, ^3^, ^4^, ^7^ Accordingly, antidepressant non‐prescription may reflect a range of clinical considerations, including diagnostic uncertainty, low current symptom burden, tolerability concerns, and clinician or patient preferences, rather than a uniform treatment pattern.^1^, ^2^, ^3^, ^7^

This issue is particularly relevant in psychiatry because patients presenting with depressed mood do not necessarily have clearly established unipolar depression at the time of care.^13^, ^14^, ^15^, ^16^, ^17^ Differential diagnosis may include bipolar depression, adjustment disorder, personality‐related affective instability, and neurodevelopmental or stress‐related conditions, all of which may influence the threshold for antidepressant prescribing.^13^, ^14^, ^15^, ^16^, ^17^, ^18^ In particular, uncertainty regarding bipolarity may lead clinicians to defer antidepressant initiation because of concerns about treatment‐emergent mood destabilization.^15^, ^16^, ^18^

Despite this clinical complexity, empirical descriptions of patients with depressive states who are not receiving antidepressants remain limited.^10^, ^11^, ^12^ Prior work has mainly focused on national prescribing trends or diagnostic coding patterns rather than on the point‐of‐care decision process that separates “prescribe” from “do not prescribe” in routine psychiatric practice.^8^, ^9^, ^10^, ^11^, ^12^ Understanding the characteristics of patients not receiving antidepressants may therefore help clarify the clinical considerations underlying non‐prescription and how psychiatrists integrate diagnostic certainty and current symptom burden in real‐world care.^1^, ^2^, ^3^, ^7^

The Japanese psychiatric context provides a useful setting for examining this question.^5^, ^16^, ^19^, ^20^, ^21^ Japanese guidance for mood disorders has emphasized structured treatment planning for MDD and bipolar disorder,^16^, ^19^, ^21^ while survey data suggest that treatment selection in Japan may differ from that in other countries and may reflect local practice patterns and resource availability.^5^, ^20^ In addition, Japanese physicians have reported challenges in the diagnosis and management of major depression in routine practice, supporting the relevance of studying prescribing decisions in ordinary care settings.^19^

Against this background, the present multicenter observational study was designed to characterize patients presenting with depressive states in Japanese psychiatric practice according to current antidepressant use status. We intentionally adopted a broad clinical definition of “depressive state” to capture the range of patients encountered in routine psychiatric practice for whom antidepressant prescribing decisions arise, including cases in which a definitive diagnosis has not yet been established. Rather than evaluating the efficacy of a specific intervention, the study aimed to describe real‐world clinical profiles and physician‐reported reasoning in routine care. We compared patients receiving antidepressants with those not receiving antidepressants using standardized case report forms across participating facilities. The objectives were to compare demographic and clinical characteristics and diagnostic certainty patterns between groups, summarize physician‐reported reasons for antidepressant non‐prescription, and perform exploratory analyses in the subgroup with high diagnostic certainty for MDD.

## METHODS

### Study design and setting

This multicenter cross‐sectional observational study used retrospectively collected routine clinical data from psychiatric facilities affiliated with the Japan Association of Psychiatric Clinics. It was based on a single observation window during March 2023 and did not include longitudinal follow‐up. No protocol‐mandated treatment procedures were introduced. The study was reported with reference to the Strengthening the Reporting of Observational Studies in Epidemiology (STROBE) statement.^22^

The protocol was approved by the Dokkyo Medical University Hospital Ethics Committee (approval no. R‐67‐5J). The study was conducted in accordance with the Declaration of Helsinki and relevant Japanese ethical guidelines.

### Participating facilities and patients

Forty facilities were invited, and 35 agreed to participate. Eligible patients were those who attended a participating facility during March 2023 and were considered by their treating psychiatrist to be under clinical management for a depressive presentation or depressive episode, including both first‐visit and follow‐up patients. This broad framework included patients with current depressive symptoms as well as patients in remission who remained under follow‐up for depressive episodes. The term “depressive state” was intentionally broad and reflected routine clinical judgment at the time of care. Eligibility was not restricted to patients with confirmed MDD. Patients with other psychiatric disorders, including anxiety disorders, could be included if they presented with a depressive state in routine clinical practice, thereby reflecting the diagnostic heterogeneity commonly encountered in everyday psychiatric practice.

Exclusion criteria were as follows: patients who had received antidepressant treatment at another institution and whose clinical information could not be adequately assessed at the participating site; patients whom the investigators judged to be inappropriate for inclusion in the study; and patients who declined participation in the study.

### Enrollment procedure

At each site, when a patient not receiving antidepressants was identified, the next consecutively seen patient receiving antidepressants was enrolled as a comparator. This enrollment procedure was intended to support contemporaneous comparison between patients with and without antidepressant treatment within routine site workflow and was not intended to estimate the prevalence of antidepressant use among all patients presenting with depressive states.

### Data collection

Physicians completed a standardized case report form based on medical records. Collected variables included age, sex, education, initial onset age category, illness duration category, current clinical state (depressed vs. remitted), and current depressive symptom severity (no symptoms, mild, severe, and very severe). In this study, “depressive state” was used as a broad eligibility concept encompassing both patients with current depressive symptoms and patients in remission who remained under follow‐up for depressive episodes in routine psychiatric practice. After enrollment, physicians categorized the patient's current clinical state at the same assessment as either “depressed” or “remitted.”

Treating psychiatrists also rated diagnostic certainty on a 5‐point scale (definite, almost definite, possible, suspected, and negative) for MDD, bipolar disorder, adjustment disorder, personality disorder, and neurodevelopmental disorders. These ratings were based on routine clinical judgment and were not derived from structured diagnostic interviews. The validity and inter‐rater reliability of this rating approach were not formally assessed in the present study. For the present analyses, definite and almost definite were combined as high diagnostic certainty, and all other ratings were categorized as not definite.

For patients not receiving antidepressants, physicians recorded reasons for non‐prescription in free‐text form. These responses were reviewed after data collection and grouped into clinically interpretable categories for descriptive analysis. Multiple reasons could be recorded for a single patient.

### Statistical analysis

Patients were categorized into antidepressant use and no‐antidepressant use groups. Continuous variables are presented as mean (SD) and were compared using Student's t‐tests. Categorical variables are presented as *n*/*N* (%) and were compared using chi‐square tests or Fisher's exact tests, as appropriate. All tests were two‐sided, and *p* < 0.05 was considered statistically significant. Statistical analyses were performed using spss Statistics version 30.0 (IBM Corp., Armonk, NY, USA). No adjustment for multiple comparisons was applied because the analyses were descriptive and exploratory; *p*‐values should be interpreted cautiously.

Analyses restricted to patients with high diagnostic certainty for MDD were prespecified as exploratory. Within this subgroup, multivariable logistic regression was used to examine factors associated with antidepressant prescription. The dependent variable was current antidepressant use (yes/no). Explanatory variables included age, sex, current clinical state, depressive symptom severity, and developmental disorder diagnostic certainty. Odds ratios (ORs) with 95% confidence intervals (CIs) are reported.

Available‐case analysis was used, and denominators are shown in the tables. No imputation was performed.

## RESULTS

### Study population

Across 35 facilities, 1342 patients presenting with depressive states at the index assessment during March 2023 were enrolled. Of these, 669 were receiving antidepressants and 673 were not receiving antidepressants, reflecting the planned comparator accrual procedure.

### Clinical characteristics according to antidepressant use status

Compared with the no‐antidepressant group, the antidepressant group was slightly older and had a lower proportion of women (Table 1). Education level and initial onset age category did not differ significantly between groups. Illness duration differed, with long‐duration illness being more frequent in the antidepressant group.

**Table 1:** Baseline characteristics of patients with and without antidepressant use.

	Antidepressant use (*N* = 669)		No antidepressant use (*N* = 673)		*p*‐value
Age, mean (SD), years	47.9	(16.3)	45.1	(17.1)	1.3 × 10^−2^
Sex, *n*/*N* (%)	*N* = 661		*N* = 670		3.4 × 10^−2^
Male	260/661	(39.3)	226/670	(33.7)	
Female	401/661	(60.7)	444/670	(66.3)	
Education, *n*/*N* (%)	*N* = 654		*N* = 658		8.3 × 10^−1^
High school or less	321/654	(49.1)	319/658	(48.5)	
College or higher	333/654	(50.9)	339/658	(51.5)	
Initial onset age, *n*/*N* (%)	*N* = 653		*N* = 663		2.6 × 10^−1^
≦30 years	249/653	(38.1)	280/663	(42.2)	
31–50 years	273/653	(41.8)	251/663	(37.9)	
≧51 years	131/653	(20.1)	132/663	(19.9)	
Illness duration, *n*/*N* (%)	*N* = 654		*N* = 660		1.2 × 10^−2^
≦11 months	70/654	(10.7)	102/660	(15.5)	
1–6 years	280/654	(42.8)	293/660	(44.4)	
≧7 years	304/654	(46.5)	265/660	(40.1)	
Current state, *n*/*N* (%)	*N* = 453		*N* = 465		2.1 × 10^−2^
Depressed	238/453	(52.5)	209/465	(45.0)	
Remitted	215/453	(47.5)	256/465	(55.0)	
Current depressive symptoms, *n*/*N* (%)	*N* = 665		*N* = 673		2.3 × 10^−3^
No symptoms	226/665	(34.0)	261/673	(38.8)	
Mild	327/665	(49.2)	345/673	(51.3)	
Severe	109/665	(16.4)	66/673	(9.8)	
Very severe	3/665	(0.4)	1/673	(0.1)	

*Note*: Values are presented as mean (SD) or *n*/*N* (%). For categorical variables, percentages were calculated using available cases for each item (denominators shown in the table); therefore, denominators vary across variables because of missing data. *p*‐values were calculated using Student's *t*‐test for continuous variables and chi‐square test or Fisher's exact test for categorical variables, as appropriate. All tests were two‐sided, and *p* < 0.05 was considered statistically significant.

Current clinical state also differed between groups. Remitted status was more frequent in the no‐antidepressant group, whereas depressed status was more frequent in the antidepressant group. Similarly, severe depressive symptoms were less frequent in the no‐antidepressant group, while the absence of current symptoms was somewhat more frequent. Overall, the no‐antidepressant group showed a profile characterized by lower current symptom burden and a higher proportion of remitted patients at the time of observation.

### Diagnostic certainty and reasons for non‐prescription

High diagnostic certainty for MDD was less frequent in the no‐antidepressant group than in the antidepressant group (Table 2). In contrast, high diagnostic certainty for bipolar disorder and adjustment disorder was more frequent in the no‐antidepressant group.

**Table 2:** Diagnostic certainty ratings according to antidepressant use status.

	Antidepressant use (*N* = 669)		No antidepressant use (*N* = 673)		*p*‐value
Lack of diagnostic certainty for antidepressant non‐use, *n*/*N* (%)	‐		409/553	(74.0)	NA
Diagnostic certainty for major depressive disorder, *n*/*N* (%)	(*N* = 665)		(*N* = 651)		1.1 × 10^−10^
Not definite	409/665	(61.5)	507/651	(77.9)	
High diagnostic certainty	256/665	(38.5)	144/651	(22.1)	
Diagnostic certainty for bipolar disorder, *n*/*N* (%)	(*N* = 636)		(*N* = 641)		2.4 × 10^−3^
Not definite	630/636	(99.1)	619/641	(96.6)	
High diagnostic certainty	6/636	(0.9)	22/641	(3.4)	
Diagnostic certainty for adjustment disorder, *n*/*N* (%)	(*N* = 642)		(*N* = 655)		7.8 × 10^−6^
Not definite	621/642	(96.7)	594/655	(90.7)	
High diagnostic certainty	21/642	(3.3)	61/655	(9.3)	

*Note*: Diagnostic certainty was rated by the treating psychiatrist on a 5‐point scale (definite, almost definite, possible, suspected, and negative). For analysis, definite and almost definite were combined as “high diagnostic certainty,” and all other ratings were categorized as “not definite.” Values are shown as *n*/*N* (%), where *N* indicates the number of available cases for each item; therefore, denominators vary across variables because of missing data. The *p*‐value is not applicable for the “lack of diagnostic certainty for antidepressant non‐use” row because it was assessed only in the no‐antidepressant group. *p*‐Values are provided for descriptive purposes and were not adjusted for multiple comparisons.

**Figure 1:**
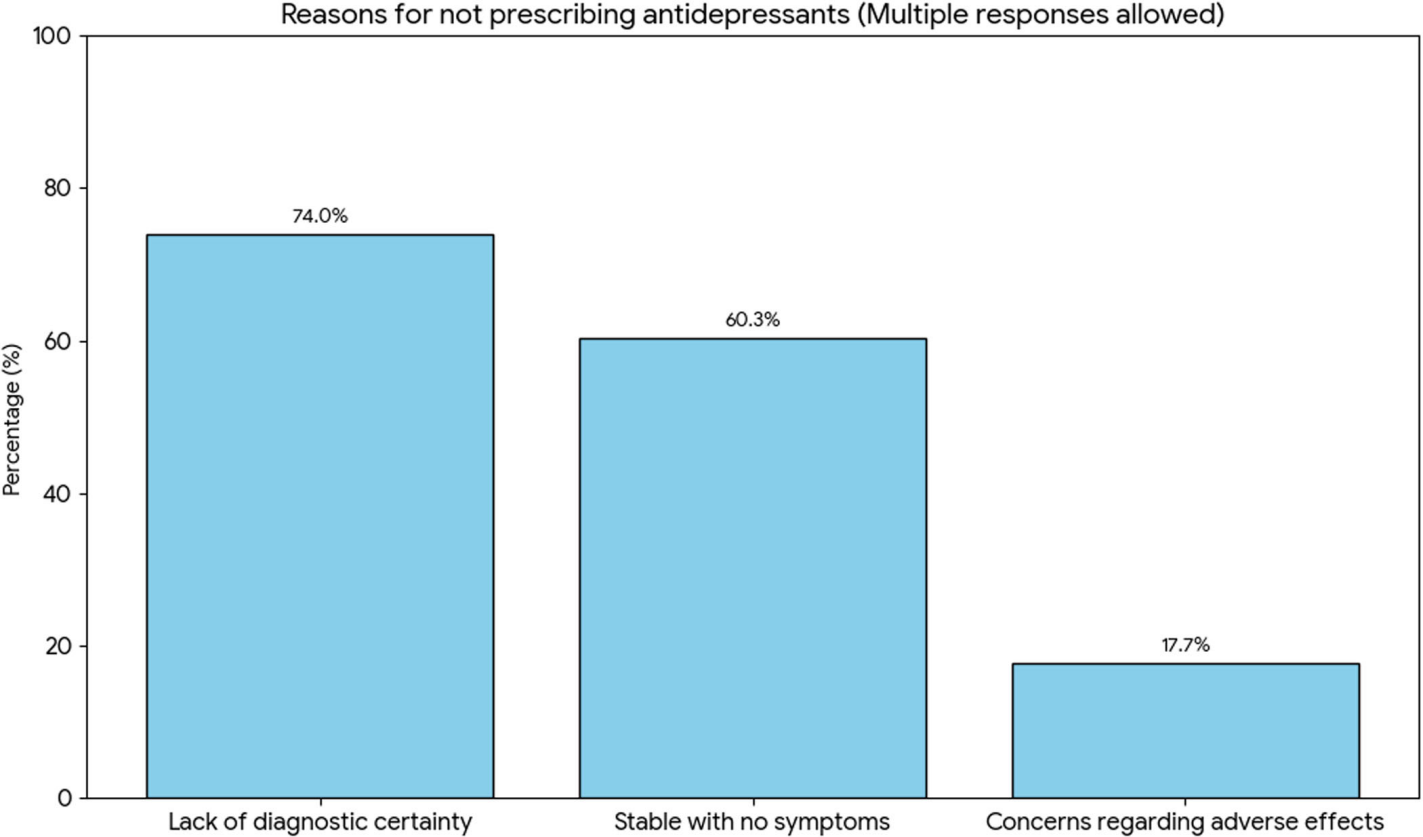
Reasons for antidepressant non‐prescription in the no‐antidepressant group. Treating psychiatrists recorded reasons for not prescribing antidepressants among patients not receiving antidepressants (available cases: *n* = 553–590, depending on item completeness). Multiple responses were allowed. Bars indicate the proportion of available cases endorsing each reason.

Among patients not receiving antidepressants, commonly reported reasons for non‐prescription included lack of diagnostic certainty, clinical stability or absence of current symptoms, and concerns regarding antidepressant adverse effects (Figure 1). Because multiple reasons could be recorded for each patient, these categories were not mutually exclusive.

These findings suggest that antidepressant non‐use in this observational sample was often accompanied by either lower confidence in an MDD diagnosis, lower current symptom burden, or both.

### Exploratory analyses in patients with high diagnostic certainty for MDD

Exploratory analyses were performed in the subgroup with high diagnostic certainty for MDD (*n* = 400) (Table 3). Within this subgroup, age, sex, education, onset age category, and illness duration were not significantly different between groups.

**Table 3:** Clinical characteristics of patients with high diagnostic certainty for major depressive disorder according to antidepressant use status.

	Antidepressant use (*N* = 256)		No antidepressant use (*N* = 144)		*p*‐value
Age, mean (SD), years	48.7	(15.1)	48.3	(17.3)	5.0 × 10^−1^
Sex, *n*/*N* (%)	*N* = 251		*N* = 144		4.4 × 10^−1^
Male	113/251	(45.0)	59/144	(41.0)	
Female	138/251	(55.0)	85/144	(59.0)	
Education, *n*/*N* (%)	*N* = 251		*N* = 142		5.8 × 10^−1^
High school or less	115/251	(45.8)	61/142	(43.0)	
College or higher	136/251	(54.2)	81/142	(57.0)	
Initial onset age, *n*/*N* (%)	*N* = 253		*N* = 143		2.3 × 10^−1^
≦30 years	74/253	(29.3)	53/143	(37.1)	
31–50 years	118/253	(46.6)	56/143	(39.2)	
≧51 years	61/253	(24.1)	34/143	(23.7)	
Illness duration, *n*/*N* (%)	*N* = 250		*N* = 143		1.6 × 10^−1^
≦11 months	30/250	(12.0)	9/143	(6.3)	
1–6 years	103/250	(41.2)	67/143	(46.9)	
≧7 years	117/250	(46.8)	67/143	(46.9)	
Current state, *n*/*N* (%)	*N* = 161		*N* = 88		2.0 × 10^−3^
Depressed	86/161	(53.4)	29/88	(33.0)	
Remitted	75/161	(46.6)	59/88	(67.0)	
Current depressive symptoms, *n*/*N* (%)	*N* = 254		*N* = 144		6.8 × 10^−3^
No symptoms	81/254	(31.9)	63/144	(43.8)	
Mild	116/254	(45.7)	67/144	(46.5)	
Severe	55/254	(21.6)	13/144	(9.0)	
Very severe	2/254	(0.8)	1/144	(0.7)	
Diagnostic certainty for bipolar disorder, *n*/*N* (%)	*N* = 245		*N* = 143		8.8 × 10^−1^
None	242/245	(98.8)	141/143	(98.6)	
Probable	3/245	(1.2)	2/143	(1.4)	
Diagnostic certainty for adjustment disorder, *n*/*N* (%)	*N* = 238		*N* = 141		5.6 × 10^−1^
None	206/238	(86.6)	119/141	(84.4)	
Probable	32/238	(13.4)	22/141	(15.6)	
Diagnostic certainty for personality disorder, *n*/*N* (%)	*N* = 246		*N* = 143		8.1 × 10^−1^
None	229/246	(93.1)	134/143	(93.7)	
Probable	17/246	(6.9)	9/143	(6.3)	
Diagnostic certainty for Developmental disorder, *n*/*N* (%)	*N* = 245		*N* = 143		6.4 × 10^−2^
None	201/245	(82.0)	106/143	(74.1)	
Probable	44/245	(18.0)	37/143	(25.9)	

*Note*: Values are presented as mean (SD) or *n*/*N* (%). Denominators (*N*) indicate the number of available cases for each item; therefore, denominators vary across variables because of missing data. *p*‐Values were calculated using Student's *t*‐test for continuous variables and the chi‐square test or Fisher's exact test for categorical variables, as appropriate. High diagnostic certainty for major depressive disorder (MDD) was defined as a rating of definite or almost definite by the treating psychiatrist. For alternative diagnoses (bipolar disorder, adjustment disorder, personality disorder, and developmental disorder), None indicates a negative rating, and Probable indicates any non‐negative rating on the same 5‐point scale (definite, almost definite, possible, or suspected). *p*‐Values are provided for descriptive purposes and were not adjusted for multiple comparisons.

In contrast, current‐state variables remained different. Remitted status was more frequent in the no‐antidepressant group, and severe symptoms were less frequent. Thus, even within a diagnostically narrower subgroup, antidepressant use status remained associated with current symptomatic burden.

### Multivariable model for antidepressant prescription

**Figure 2:**
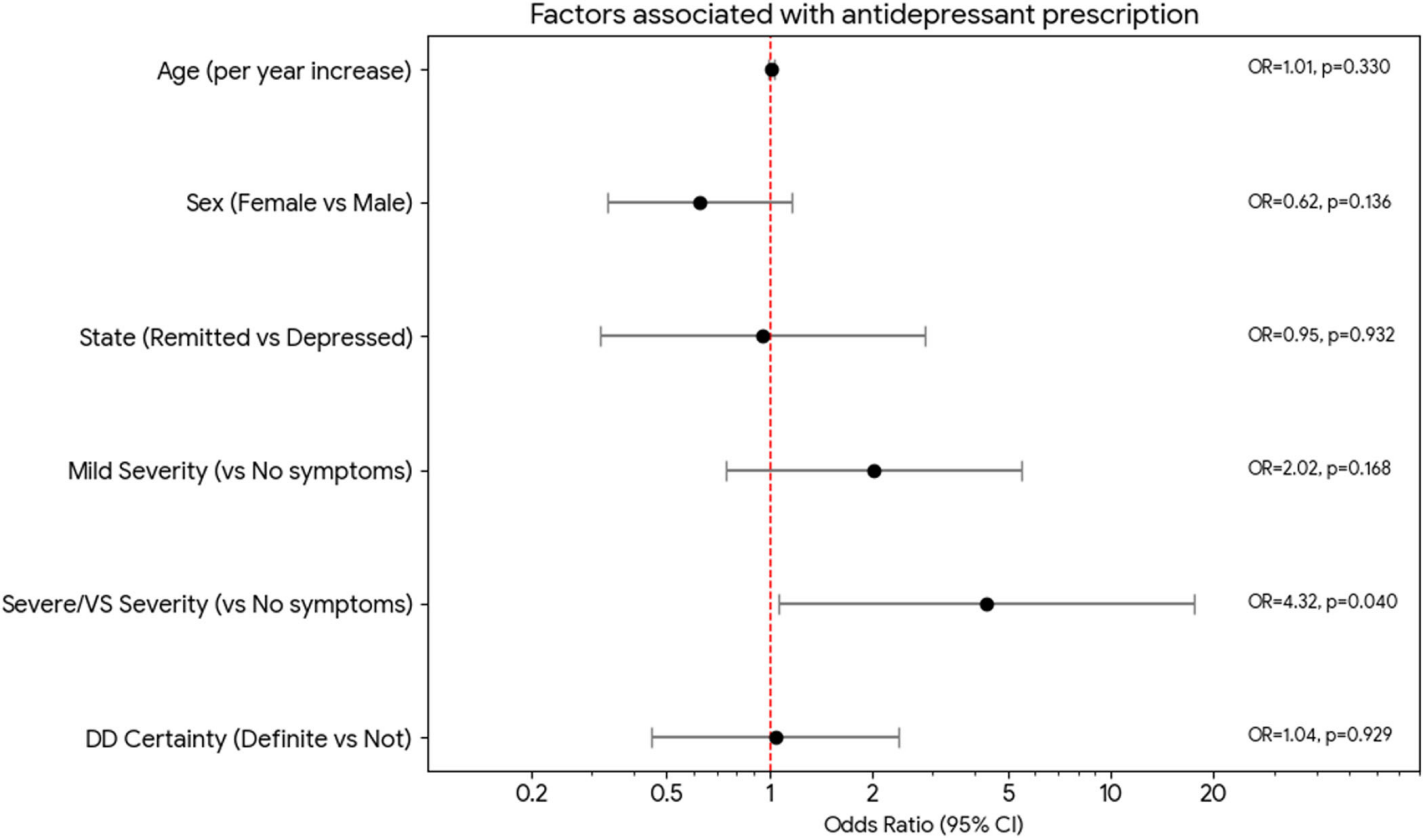
Factors associated with antidepressant prescription in patients with high diagnostic certainty for major depressive disorder (MDD). Forest plot of adjusted odds ratios (ORs) with 95% confidence intervals (CIs) from a multivariable logistic regression model examining factors associated with current antidepressant use within the subgroup with high diagnostic certainty for MDD (*n* = 400). The model included age, sex, current clinical state, depressive symptom severity (reference: no symptoms), and developmental disorder diagnostic certainty. Severe/very severe symptoms were associated with higher odds of antidepressant prescription (OR 4.317, 95% CI 1.067–17.462). DD, developmental disorder.

In the multivariable logistic regression model restricted to patients with high diagnostic certainty for MDD, severe or very severe symptoms (vs. no symptoms) were associated with higher odds of antidepressant prescription (Figure 2). Mild symptoms showed a positive but non‐significant association. Age, sex, current clinical state, and developmental disorder diagnostic certainty were not statistically associated.

Taken together, the adjusted analysis suggests that among patients with high diagnostic certainty for MDD, current symptom severity was the factor most clearly associated with antidepressant prescription in this dataset. However, the wide CI for the symptom severity estimate (OR = 4.317, 95% CI 1.067–17.462) reflects the limited sample size in the exploratory subgroup and should be interpreted with caution.

## DISCUSSION

In this multicenter observational study of routine psychiatric practice in Japan, antidepressant use status among patients presenting with depressive states was associated with both diagnostic certainty and current symptom burden.^1^, ^2^ Compared with patients receiving antidepressants, those not receiving antidepressants were less likely to have high diagnostic certainty for MDD and were more likely to be remitted or less symptomatic at the time of observation. These findings are broadly consistent with the idea that antidepressant prescribing in routine care is shaped not only by diagnosis labels but also by uncertainty management and perceived illness intensity.^1^, ^2^, ^3^, ^7^, ^19^

One of the most notable findings was the lower level of MDD diagnostic certainty in the no‐antidepressant group. This pattern is clinically plausible because depressive presentations in everyday practice frequently require differentiation from bipolar disorder, adjustment disorder, and other overlapping conditions.^13^, ^14^, ^15^, ^16^, ^17^ When bipolarity is considered possible, clinicians may reasonably adopt a more cautious approach to antidepressant initiation because of concerns regarding mood destabilization or switch risk.^15^, ^16^, ^18^ Likewise, when symptoms are interpreted as context‐related or adjustment‐related, observation, supportive management, or psychosocial intervention may be prioritized over immediate antidepressant treatment.^1^, ^2^, ^4^, ^7^

The greater frequency of high diagnostic certainty for bipolar disorder and adjustment disorder in the no‐antidepressant group is therefore consistent with risk‐aware prescribing behavior.^13^, ^14^, ^15^, ^16^, ^18^ This interpretation is also compatible with prior work indicating that treatment choices in depression vary across settings and are influenced by clinical judgment beyond symptom presence alone.^11^, ^12^, ^19^, ^20^ Importantly, however, the present data are cross‐sectional and observational; they indicate associations with prescribing status but cannot establish temporal sequence or clinical intent in individual cases.

Current symptom burden was another recurring correlate of antidepressant prescription. In both the full cohort and the exploratory subgroup with high diagnostic certainty for MDD, remitted status and lower symptom severity were more common in the no‐antidepressant group. In the multivariable model, severe or very severe symptoms were associated with higher odds of antidepressant prescription. Although this does not establish causality, it is consistent with guideline‐based and meta‐analytic evidence indicating that antidepressants are principally used for clinically significant depressive states and that treatment intensity tends to increase with greater symptom burden.^1^, ^2^, ^3^, ^4^, ^6^, ^7^

The present findings also fit with the broader literature suggesting that antidepressant prescribing is heterogeneous and sensitive to clinical context.^8^, ^9^, ^10^, ^11^, ^12^ In Japanese practice, treatment selection may differ from that in other countries and may be influenced by local service structure, access to psychotherapy, and physician preferences.^5^, ^19^, ^20^ From this perspective, the observed association between lower current symptom burden and antidepressant non‐use should not be viewed solely as undertreatment; rather, it may in many cases reflect individualized clinical balancing of expected benefit, tolerability concerns, and the need for further diagnostic clarification.^1^, ^2^, ^3^, ^7^

An additional strength of this study is the inclusion of physician‐reported reasons for non‐prescription. Administrative and claims databases can identify whether a medication was prescribed, but they rarely capture the contemporaneous clinical reasoning underlying non‐prescription.^8^, ^9^, ^10^, ^11^, ^12^ In the present study, lack of diagnostic certainty and clinical stability or absence of current symptoms were frequently recorded as reasons for not prescribing antidepressants. These observations support the view that non‐prescription often occurred in the context of explicit clinical judgment rather than simple omission of care.^1^, ^2^, ^3^, ^7^

The findings should be interpreted in light of several limitations. First, the retrospective cross‐sectional observational design does not allow causal inference. Second, diagnostic certainty ratings were based on routine clinical judgment rather than structured diagnostic interviews. Their validity and inter‐rater reliability were not formally evaluated in this study, and the ratings may therefore be subject to inter‐clinician variability. Third, the intentionally broad definition of “depressive state” improved the relevance of the study to routine psychiatric practice, where treatment decisions often must be made before diagnostic formulation is fully stabilized. At the same time, this approach may reduce reproducibility compared with studies restricted to patients diagnosed using structured criteria, and the external validity of the findings should therefore be interpreted in relation to similar real‐world clinical settings. Fourth, reasons for non‐prescription were derived from physician‐recorded free‐text responses and categorized after collection; the depth of documentation may have varied across sites. Fifth, the study captured treatment status at a single observation window and did not evaluate longitudinal outcomes such as later antidepressant initiation, relapse, hospitalization, or symptom trajectories. Sixth, participating facilities belonged to one professional network and may not represent all psychiatric settings in Japan. Seventh, missing data were handled by available‐case analysis without imputation. Sensitivity analyses were not performed; therefore, the robustness of the findings to alternative assumptions (e.g., missing data mechanisms or site‐level clustering) could not be evaluated. Eighth, site‐level clustering and the comparator accrual structure were not explicitly modeled in the analyses, and the findings should therefore be interpreted as descriptive and exploratory. Ninth, we did not collect detailed information on non‐pharmacological treatments, such as cognitive behavioral therapy, supportive psychotherapy, or neuromodulation interventions including repetitive transcranial magnetic stimulation. Therefore, we could not evaluate how these treatment options may have contributed to antidepressant non‐prescription in routine practice. Finally, the exploratory subgroup model was limited in size, and several estimates had wide CIs, limiting the precision of effect estimates.

Despite these limitations, the study has several strengths. It included a relatively large multicenter sample, used a pragmatic enrollment approach that reflected routine site workflow, and incorporated physician‐reported reasons for non‐prescription that are rarely available in administrative data sources.^8^, ^9^, ^10^, ^11^, ^12^ The combination of full‐cohort comparisons and exploratory analyses in a subgroup with high diagnostic certainty for MDD provides a clinically informative description of prescribing‐associated factors under different levels of diagnostic certainty.

Future prospective studies with longitudinal follow‐up, standardized symptom scales, detailed information on non‐pharmacological treatments, and patient‐reported decision factors may help clarify how clinicians and patients negotiate antidepressant treatment in heterogeneous depressive presentations.^1^, ^2^, ^3^, ^7^

## CONCLUSION

In this multicenter observational sample, antidepressant non‐use among patients presenting with depressive states was associated with lower diagnostic certainty for MDD and with lower current symptom burden at the time of observation. Among patients with high diagnostic certainty for MDD, greater symptom severity was associated with antidepressant prescription. These findings may help contextualize conservative treatment decisions under diagnostic uncertainty in routine psychiatric practice.

## AUTHOR CONTRIBUTIONS

Hiroyuki Muraoka conducted the data analysis and drafted the first version of the manuscript. Norio Yasui‐Furukori supervised the project, contributed to the study design, and coordinated data acquisition across participating facilities. Ryota Hashimoto contributed to the statistical analysis and interpretation of the data. Kentaro Fukumoto, Fumitoshi Kodaka, Kazutaka Ohi, and Ken Inada contributed to the interpretation of the data and critically revised the manuscript for important intellectual content. Data collection was supported by participating clinicians and facilities listed in the Acknowledgments. All authors reviewed and approved the final manuscript.

## CONFLICT OF INTEREST STATEMENT

The authors declare no conflicts of interest.

## ETHICS APPROVAL STATEMENT

This study was approved by the Dokkyo Medical University Hospital Ethics Committee (approval no. R‐67‐5J) and was conducted in accordance with the Declaration of Helsinki and relevant ethical guidelines in Japan.

## PATIENT CONSENT STATEMENT

Informed consent was obtained using an opt‐out approach approved by the ethics committee.

## CLINICAL TRIAL REGISTRATION

Clinical trial registration: Not applicable.

## Data Availability

The data are not publicly available due to privacy and ethical restrictions. The opt‐out consent procedure did not include consent for public release of individual‐level data. Data may be available from the corresponding author upon reasonable request and with permission from the relevant ethics committee.
